# Trailing TRAIL Resistance: Novel Targets for TRAIL Sensitization in Cancer Cells

**DOI:** 10.3389/fonc.2015.00069

**Published:** 2015-04-02

**Authors:** Rachana Trivedi, Durga Prasad Mishra

**Affiliations:** ^1^Cell Death Research Laboratory, Division of Endocrinology, CSIR-Central Drug Research Institute, Lucknow, India

**Keywords:** TRAIL, cancer, apoptosis, TRAIL-resistance, DR4, DR5

## Abstract

Resistance to chemotherapeutic drugs is the major hindrance in the successful cancer therapy. The tumor necrosis factor-related apoptosis-inducing ligand (TRAIL) is a member of the tumor necrosis factor (TNF) family of ligands, which initiates apoptosis in cancer cells through interaction with the death receptors DR4 and DR5. TRAIL is perceived as an attractive chemotherapeutic agent as it specifically targets cancer cells while sparing the normal cells. However, TRAIL therapy has a major limitation as a large number of the cancer develop resistance toward TRAIL and escape from the destruction by the immune system. Therefore, elucidation of the molecular targets and signaling pathways responsible for TRAIL resistance is imperative for devising effective therapeutic strategies for TRAIL resistant cancers. Although, various molecular targets leading to TRAIL resistance are well-studied, recent studies have implicated that the contribution of some key cellular processes toward TRAIL resistance need to be fully elucidated. These processes primarily include aberrant protein synthesis, protein misfolding, ubiquitin regulated death receptor expression, metabolic pathways, epigenetic deregulation, and metastasis. Novel synthetic/natural compounds that could inhibit these defective cellular processes may restore the TRAIL sensitivity and combination therapies with such compounds may resensitize TRAIL resistant cancer cells toward TRAIL-induced apoptosis. In this review, we have summarized the key cellular processes associated with TRAIL resistance and their status as therapeutic targets for novel TRAIL-sensitizing agents.

## Introduction

Pre-existing or acquired resistance to chemotherapy is a major obstacle in effective cancer therapy, as it often leads to the therapy failure and the disease relapse (1). Therefore, there is an ever increasing need for development of safe drugs and novel therapeutic strategies for targeting these chemo-resistant cancer cells. It is imperative that these chemotherapeutic agents or strategies should selectively target the cancer cells in an irreversible manner without harming the normal cells (2). However, poor tolerance and chemotherapy associated side effects are still major hurdles in therapeutic targeting of chemo-resistant cancer cells. Chemotherapeutic drugs can target cancer cells through inhibition of cellular proliferation and survival or induction of cell cycle arrest and apoptosis. Among these processes, apoptosis is an evolutionarily conserved and the most widely studied cellular response, essential for maintenance of tissue homeostasis and removal of unwanted cells (3). Apoptosis is triggered by either the intrinsic or extrinsic stimuli. The intrinsic pathway of apoptosis includes cellular damage brought about by stress, ultraviolet (UV) or ionizing radiation, or oncogene activation. On the other hand, the extrinsic pathway of apoptosis is triggered by the binding of extracellular ligands to specific transmembrane receptors resulting in caspase activation and cell death (2, 4). The tumor necrosis factor-related apoptosis-inducing ligand (TRAIL) belongs to the group of chemotherapeutic agents selectively targeting a wide variety of cancer cells without affecting the normal cells (5–8). The therapeutic potential of TRAIL is attributed to its receptor expression in a variety of tissues like lymphocytes, spleen, thymus, ovary, prostate, colon, intestine, and placenta compared to the restricted and transient expression of other ligands of the TNF family (8). Therefore, TRAIL is considered as a promising and effective anticancer agent under clinical investigation (9, 10). The therapeutic usage of other members of the TNF super family like Fas/FasL and TNFα/TNFR1 are limited due to their severe side effects including lethal septic shock like responses (11). The Fas/FasL and TNFα/TNFR1 are known to activate the oncogenic NF-κB pathway while TRAIL shows weak effects on NF-κB activation, and is therefore considered safe as a therapeutic agent. It also plays an important role in the natural killer cell mediated immunosurveillance against the rapidly growing and metastatic cancer cells (8). Pre-clinical studies have shown that administration of the soluble form of the recombinant TRAIL in mice and non-human primates suppressed the proliferation of TRAIL sensitive human tumor xenografts, with no apparent systemic toxicity underscoring the potential utility of rhTRAIL *in vivo* (6, 7). However, the major limitation of the TRAIL therapy is development of TRAIL resistance through a variety of mechanisms in cancer cells. Therefore, to enhance the TRAIL mediated apoptotic effect, the combination of TRAIL along with novel TRAIL sensitizing agents possibly represents the best clinical option (Table 1).

**Table 1:** **Small molecule with TRAIL sensitization ability**.

Target	Drug	Tissue (cell line)	TRAIL-sensitizing mechanism
ER-stress induction	Verrucarin A	Liver (Hep3B) (118)	eIF2α/CHOP-dependent DR5 induction via ROS generation
	Monensin	Brain (U251MG), U87MG (119)	CAAT/enhancer binding protein homologous protein (CHOP) dependent DR5 induction
	Nigericin	
	Salinomycin	
	Narasin	
	Lasalocid A	
	Medicarpin	Blood (K562, U937) (120)	CHOP dependent DR5 up-regulation
	Diallyl trisulfide (DATS)	Skin (A375) (72)	CHOP mediated DR5 up-regulation and c-FLIP down-regulation
	Oligomycin A	Cervical (HeLa) (121)	Disrupting the adaptation to ER-mediated death pathway
	Tunicamycin	Skin (Mel-RM, MM200) (122)	DR5 up-regulation via the unfolded protein response
	15-deoxy-Δ-12,14-prostaglandin J2 (15dPGJ(2))	Colon (HCT116) (123)	CHOP dependent DR5 up-regulation via ROS generation
	Dibenzylideneacetone (DBA)	Colon (HCT116, HT29) (124)	Down-regulation of cell survival proteins and up-regulation of death receptors via activation of ROS and CHOP mediated pathways
	5,7-dimethoxyflavone (DMF)	Liver (Hep3B, Huh-7, and Hep G2) (125)	ROS-stimulated ER-stress triggering CHOP-mediated DR5 up-regulation
Metastasis	Neobavaisoflavone (NBIF)	Brain (U373MG) (126)	DR5 up-regulation
	4,5-dimethoxy-2-nitrobenzaldehyde (DMNB)	Metastatic colon (KM12L4A) and prostate (PC3-MM2) (127)	Up-regulation of DR5 and inactivation of DNA-dependent protein kinase (DNA-PK)/Akt, a pathway required for cancer cell metastasis
	MG132	Head and neck (128)	Stabilizing tBid and Bik
		Liver (HepG2)	Modulating the interaction of FADD and the TRAIL death receptors
	Bortezomib (VELCADE)	Head and neck (129)	A caspase-dependent, E6-independent mechanism
		Kidney (Caki1,UO-3, ACHN) (130)	Increased in activation of caspase-8 in the death-inducing signaling complex
		Brain (U373MG) (131)	p53-independent DR5 up-regulation
		Brain (132)	PKCε-dependent down-regulation of AKT and XIAP expressions
		Brain (U373, LNZ308) (133)	Inhibiting the NF-κB signaling pathway
Proteosome inhibition		Oesophagus (established cell lines KE4, TE8, TE9) (134)	Activation of both extrinsic and intrinsic apoptosis pathways
		Prostate (LNCaP, PC3) (135)	Stabilization of the TRAIL receptor DR5 mRNA through the 3′-untranslated region
		Lung (H460, A549, SW1573, H292, H1299, and H322) (80)	Increased activation of caspase-8-mediated as well as caspase-9-mediated apoptosis
		B-Cell (HRC57) (136)	Blocking bax degradation
		Thyroid (8305C, ARO, and KAT4) (137)	Down-regulation Bcl-2 and Bcl-X_L_, and the up-regulation of p21 and SMAC/Diablo
	NPI-0052	Prostate (PC3) human non-Hodgkin’s B cell lymphoma (B-NHL) (Ramos) (82)	Inhibits the transcription repressor Yin Yang 1 (YY1), which negatively regulates DR5 transcription
Heat shock proteins	Geldanamycin	Prostate (LNCaP, DU145) (104)	Hsp90 inhibition and increased activation of caspase-3, caspase-7, and their substrate poly (ADP-ribose) polymerase
	17-AAG	Brain ((U87MG, LN229, and U251) (138)	HSp90 inhibition and down-regulating survivin through proteasomal degradation
	LY30	Cervical (HeLa) (139)	Sustained phosphorylation of Hsp27 and inhibition of its protective functions
Autophagy	Pifithrin (PFT)-μ	Pancreatic (MiaPaca-2, Panc-1) (140)	Inhibits HSP70-induced stabilization of lysosomal membrane permeabilization, resulting in increased cell death
	HDAC Inhibitor		
	MS-275	Breast cancer (MDA-MB-231) (141)	Activation of downstream caspase-3, which can be activated by both extrinsic and intrinsic pathways
	Depsipeptide	Lymphoid (Jurkat) (142)	By facilitating formation of an active death-inducing signaling complex (DISC), leading to the rapid activation of caspase-8
	NaB and SAHA	Brain (SHEP) (143)	Change in the equilibrium of pro-to anti-apoptotic molecules that lowers the cell death threshold and strongly favors apoptosis
Epigenetic modulation	LGP1, a HDAC inhibitor analog of FR235222	Blood (Jurkat,HL60), Breast (MCF-7) (144)	Activate the DR5 gene through p53-independent regulation
	TSA	Myeloid (U937) (145)	Up-regulation of TRAIL-R1 receptor
	SIRT1 inhibitor		
	Amurensin G	Blood (Jurkat,HL60), Breast (MCF-7) (144)	Activate the DR5 gene through p53-independent regulation
	Valproic acid (VPA)	Myeloid (K562) (146)	Up-regulation of c-Myc and DR5 surface expression and the down-regulations of c-FLIP and Mcl-1
	DNA demethylation		
	Decitabin	Lung (H69, H82 H1417 H2171, and U1906 (147)	Efficient restoration of caspase-8
		Brain (T98G, U87MG, U251, and TB10) (148)	Up-regulation of TRAIL receptor-1 and caspase-8, down-regulation of PED/PEA-15
		Lung (H69, H82 H1417 H2171, and U1906 (147)	Efficient restoration of caspase-8
		Brain (SH-SY5Y, LAN1, Kelly, and D283Med) (149, 150)	Re-express caspase-8 in cancer cells lacking caspase-8
		Skin (MEWO, MML-1) and Blood (Jurkat, CEM) (150)	Increasing expression level of caspase-8
	Glycolysis inhibitor		
Metabolic pathways	2-Deoxy-d-Glucose	Skin (MelRM, Mel200, Mel-CA, and Mel-MC) (66)	XBP-1-mediated up-regulation of TRAIL-R2
		Blood (U937,Jurkat) and Cervical (HeLa) (151)	AMPK activation and mammalian target of rapamycin (mTOR) inhibition leading to Mcl-1 decrease
	Glyoxalase pathway		
	Methylglyoxal (MG)	Colon (SW480) (152)	Suppresses expression of antiapoptotic factors, X-linked inhibitor of apoptosis protein (XIAP), survivin, cIAP1, Bcl-2, and Bcl-xL
	Nucleoside transport inhibitor		
	Dipyridamole	Colon (SW480), Bone (MG63), Prostate (DU145) (153)	CHOP dependent DR5 up-regulation
	Thymidylate synthase inhibitor		
	Trifluorothymidine (TFT)	Lung (A549, H292, H322, and H460) (154)	Increased the expression of p53 and p21/WAF1, and p53-dependent DR5 expression
	Mitochondrial pyrimidine biosynthesis		
	Doxorubicin and Brequinar	Lung (U1690), Breast (MC7), Prostate (LNCaP) (155)	Inhibition dihydroorotate dehydrogenase (DHODH) and down-regulation of c-FLIP_L_ as well as by mitochondrial depolarization
Protein synthesis	Cycloheximide	Prostate (PC3) (156)	JNK activation and c-FLIP down-regulation
		Colon (KM12C, KML4A, and KM20) (157)	JNK activation and c-FLIP down-regulation
	Anisomycin	Prostate (PC3) (156)	JNK activation
	Salubrinal	Liver (HepG2) (67)	Inhibition of eIF2α dephosphorylation

## TRAIL, Its Receptors and Apoptotic Pathway

**Figure 1:**
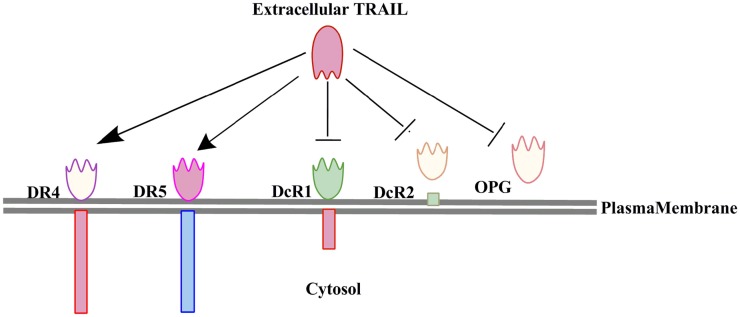
**Interaction of TRAIL with its five receptors (two agonostic receptors: DR4, DR5; and three antagonistic receptors: DcR1, DcR2, and OPG)**. Only the two agonistic receptors DR4 and DR5 can transduce the TRAIL induced cell death signaling.

TRAIL is a member of the TNF-related proteins having structural and functional similarity with CD95L. TRAIL is a 20 kDa protein encoded by a gene with five exons and three introns located on the chromosome 3 (12–14). TRAIL is mainly expressed on the cells of the immune system and plays critical roles in T-cell homeostasis and NK or T-cell mediated killing of virally and oncogenically transformed cells (15, 16). TRAIL is a type II transmembrane protein with an extracellular domain which can be cleaved to form its biologically active soluble form (17). Initially TRAIL was identified and cloned based on the sequence homology of its extracellular domain with CD95L (28% homology) and TNFα (23% homology) (17). However, its extracellular carboxy terminal portion is proteolytically cleaved from the cell surface in a vesicle associated or soluble form (17, 18). Previous studies have also shown that TRAIL interacts with two agonistic receptors i.e., (1) TRAIL-R1 (DR4) and (2) TRAIL-R2 (DR5/TRICK2/KILLER) (19–21), and three antagonistic receptors i.e., (1) TRAIL-R3 (DcR1/TRID/LIT), (2) TRAIL-R4 (DcR2/TRUNND), and a soluble receptor i.e., osteoprotegerin (OPG) (22, 23). OPG was identified initially as a receptor for the receptor activator of nuclear factor kappa-B ligand (RANKL) (24) (Figure 1). TRAIL-R1 and TRAIL-R2 are type I transmembrane proteins sharing a sequence homology of 58% (25) with a cytoplasmic or death domain which recruits apoptosis signaling molecules for the induction of cell death (26). The TRAIL-R1 and TRAIL-R2 expression is regulated by p53 and the TRAIL-R2 gene promoter has a p53 responsive element (27). The TRAIL receptors TRAIL-R1 and TRAIL-R2 not only trigger apoptosis in TRAIL-sensitive cells but also activate survival pathways in tumor cells that resist the induction of cell death upon exposure to TRAIL (28). Post-translational modifications such as glycosylation and palmitoylation of DR4 and DR5 death receptors are also important regulators of TRAIL induced signaling (29). There exists a correlation between the expression of glycosylation initiating enzyme polypeptide *N*-acetylgalactosaminyltransferase 14 (GALNT14) and sensitization toward TRAIL mediated apoptosis in different cancers like pancreatic carcinoma, lung cancer, and malignant melanoma (30). O-glycosylation enhanced ligand-induced clustering of DR4 and DR5, which mediated recruitment and activation of apoptosis-initiating protease caspase-8 (31). TRAIL binds to its receptor as a homotrimer form, which is biologically much more active than the trimeric form. TRAIL-R3 and TRAIL-R4 lack the functional death domain (DD), and therefore are unable to transmit the apoptotic signals induced by binding to TRAIL ligands (23, 32). Therefore, TRAIL-R3 and TRAIL-R4 are believed to be competitive inhibitors regulating TRAIL-induced apoptosis (20). OPG is the only soluble receptor of TRAIL with lower binding affinity as compared to the other death receptors (24).

**Figure 2:**
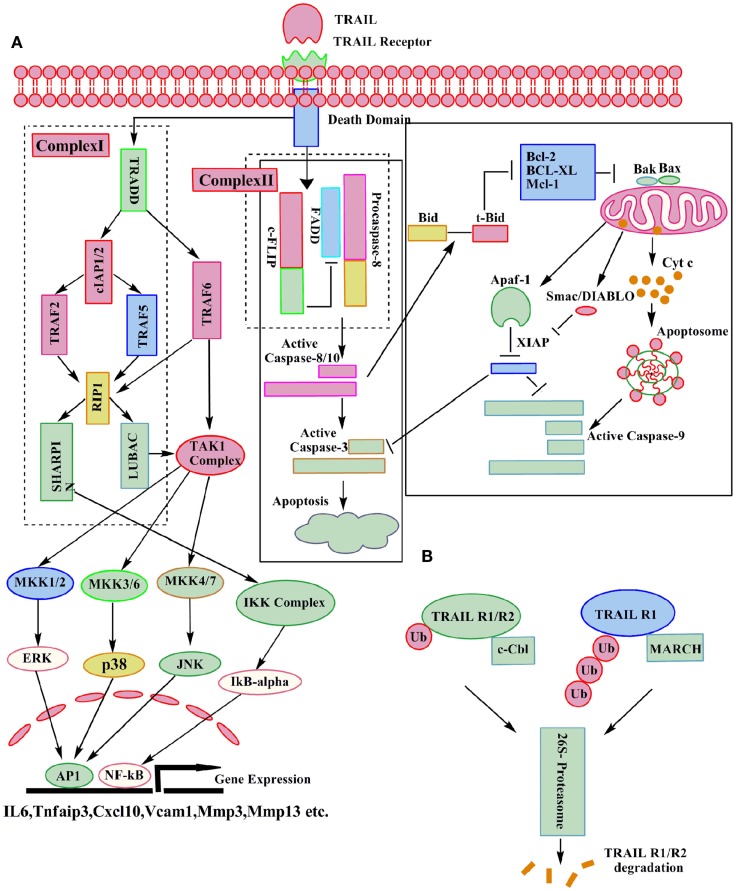
**Molecular details of canonical and non-canonical TRAIL signaling**. Following TRAIL binding to its death receptors, the DISC can be formed which results in caspase-3 activation and apoptosis. A secondary complex can also be formed after TRAIL receptor activation, leading to the activation of various kinases and the induction of direct or indirect non-apoptotic responses as indicated **(A)**. The ubiquitin–proteasome system can assist in the degradation of TRAIL-Rs **(B)**.

Depending upon the stimuli, either the extrinsic or the intrinsic pathways of apoptosis are activated (33) in a cancer cell. The cross talk between these two pathways is mediated through the truncation of the pro apoptotic protein Bid (34). The extrinsic pathway is mediated through the binding of TRAIL to its two death receptors DR4 (TRAIL R1) and DR5 (TRAIL R2). Binding of TRAIL to its receptors leads to the trimerization of receptors and formation of the death inducing signaling complex (DISC) (25). An adaptor protein FADD translocates to the DISC, and interacts with the DD, and facilitates the recruitment of procaspase-8/10 through interaction of their respective death effector domains (DED). Self activation of these initiator caspases (35) by DISC is required for the execution of apoptosis via the extrinsic pathway. In some cell types, type I activation of caspase-8 is sufficient for the subsequent activation of the effector caspase-3 and execution of apoptosis (2). But in other cell types, type II involvement of the mitochondrial pathway (intrinsic pathway) is required (4). In case of the intrinsic pathway, activation of caspase-8 leads to cleavage of Bcl-2 inhibitory BH3-domain interacting protein (Bid) (36). Subsequently, the truncated Bid interacts with Bax and Bak and induces their oligomerization in the mitochondrial membrane, which leads to the loss of the mitochondria membrane potential and ultimately release of cytochrome *c* (37) and Smac/Diablo (38) (Figure 2). At the DISC, activation of caspase-8 and caspase-10 can be inhibited by cellular FLICE-like inhibitory protein (c-FLIP) (39). Type II cells also require the inactivation of intracellular apoptosis inhibitors, such as X-linked inhibitor of apoptosis protein (XIAP), which directly inhibits the effector caspase activity (40). The paradigm-changing model for DISC assembly and structure indicated that FADD is substoichiometric and procaspase-8 is recruited, not only through an interaction with FADD but also by interacting with itself. The DED chain assembly model also presents the intriguing possibility that only a small amount of DISC is required for activation of large amounts of caspase-8 (41). Like caspase-8 and caspase-10, c-FLIP also has two DEDs, and has 13 discrete splice variants, and three of which are expressed as proteins: the 26 KDa short form (c-FLIP_S_), the 24 KDa form of c-FLIP (c-FLIP_R_), and the 55 KDa long form (c-FLIP_L_) (42, 43). The C-terminus of c-FLIP_S_ is smaller than that of c-FLIP_L_ and very much similar to the caspase-8 and caspase-10 structure, but this region of c-FLIP_L_ does not contain a functional caspase domain, which is due to substitution of several amino acids, mainly the crucial cysteine residue in the catalytic domain which is necessary for the catalytic activity of caspases (43, 44). In humans, single nucleotide polymorphism defines the production of c-FLIP_S_ or c-FLIP_L_ in a three′ splice site of the c-FLIP gene. An intact splice site directs production of c-FLIP_S_, but the splice-dead variant results in production of c-FLIP_R_. Both c-FLIP_L_ and c-FLIP_S_ isoforms are short-lived proteins and are largely degraded by the ubiquitin–proteasome degradation system. Levels of c-FLIP_L_ and c-FLIP_S_ are regulated by JNK activation via the E3 ubiquitin ligase Itch and also through phosphorylation. The protein kinase C (PKC) phosphorylation at the serine 193 (S193) residue of c-FLIP_S_ inhibits its polyubiquitination, stabilizes c-FLIP_S_ levels, and increases cell survival (45, 46). c-FLIP isoforms are reported to be overexpressed in pancreatic cancer, where as very low or no expression is found in normal pancreatic ducts (47). c-FLIP protein enhances the anti-apoptotic activity of Akt by modulating GSK3β activity and thus induces resistance to TRAIL (48). High-grade prostatic intraepithelial neoplasia (HGPIN) and prostate cancer are found to express high level of c-FLIP as compared to normal prostate epithelium (47). The naturally occurring differences in the levels or states of proteins regulating receptor-mediated apoptosis are the primary causes of cell-to-cell variability in the timing and probability of death (49).

## Protein Synthesis and TRAIL Resistance

Many disease conditions are attributed to failure in synthesis of a specific active protein (50). Such conditions generally involve a mutation of the gene encoding the protein, leading to an altered protein level or activity (51). Protein translational control is an important strategy in regulation of eukaryotic gene expression. Interestingly, dysregulated translation has now been linked to multiple human cancers (52). A major target of translational control is the eukaryotic translation initiation factor 4E (eIF4E), which interacts with the 7-methylguanosine cap structure located at the 5′ untranslated regions of cellular messenger RNA (mRNA) and transfers these mRNA to the eIF4F translation initiation complex, an assembly of the cap-binding protein eIF4E, the RNA helicase eIF4A, and the scaffolding protein eIF4G (53, 54). Availability of eIF4E is the determining factor for the assembly of eIF4F. As eIF4E is scarce among the initiation factors involved in the eIF4F complex, eIF4E is the rate-limiting factor for cap-dependent translation initiation (54). Several human cancers exhibit inadequate eIF4F activation. Inhibitor of eIF4E/eIF4G interaction can act as a TRAIL sensitizer by down-regulating the levels of cyclin D1 and hypoxia-inducing factor-1α (HIF-1α), and both of which follow the cap-dependent translation regulation mechanism (55). Inhibitors of the eIF4E/eIF4G increase TRAIL-induced apoptosis through the up-regulation of DR5 and inhibition of c-FLIP, independent of inhibition of cap-dependent protein translation (56). JNK signaling induces apoptosis by inducing secretion of death ligands to promote release of cytochrome *c* from mitochondria to cytosol or by posttranslational modification phosphorylation of downstream pro-apoptotic proteins (57). It has been also reported that JNK activation up-regulates DR5 expression, which leads to apoptosis in cancer cells through caspase-8 activation (58). JNK has also been reported to up-regulate the expression of CHOP via an AP-1 binding site in promoter region in HeLa cells. This JNK dependent CHOP expression leads to DR5 up-regulation and induces TRAIL mediated apoptosis (58).

## ER-Stress and TRAIL Resistance

Endoplasmic Reticulum (ER) is a key intracellular organelle involved in the regulation of protein synthesis, proper folding of newly synthesized proteins and regulation of the intracellular calcium levels (59, 60). The malfunctioning of the aforementioned processes leads to the cellular stress response known as ER stress. ER stress induces signaling pathway which is known as the “unfolding protein response” (UPR). The prolonged and severe ER stress leads to apoptosis (61, 62). ER stress induces activation of the intrinsic apoptotic pathway (63) through the DR5 death receptors (64). DR5 up-regulation by ER stress inducing agents has been suggested to play a crucial role in the sensitization of TRAIL resistant cells (65, 66). Activation of TRAIL receptors induces translocation of pro-apoptotic Par-4/GRP78 complex to the cell surface of cancer cells. Combination of salubrinal and TRAIL leads to dephosphorylation of eIF2-α increased expression of Bim, a CHOP-regulated proapoptotic protein in hepatoma cells for TRAIL sensitization (67). Par-4 is spontaneously secreted by normal and cancer cells in culture and also by Par-4 transgenic mice that are resistant to spontaneous tumors. Par-4 induces apoptosis by binding to glucose regulated protein-78 (GRP78) which results in ER-stress and activation of the FADD/caspase-8/caspase-3 pathway (68). Thus Par-4 activates extrinsic pathway involving cell surface GRP78 receptor for apoptosis induction (69). An increasing number of reports have also demonstrated that inducers of ER stress also sensitize glioblastoma, colon cancer, breast cancer, melanoma, and hepatoma cells to TRAIL induced apoptosis (70–72). ER stress inducers provide a promising option for sensitizing TRAIL resistant cells as ER stress alters the cellular levels of different apoptosis-related proteins responsible for TRAIL resistance, including a decrease in the levels of FLIP and Mcl-1 and the up-regulation of DR5 (70–72).

## The Ubiquitin–Proteasome Pathway and TRAIL Resistance

Protein stabilization is a key regulatory mechanism required for the control of cellular development, cell growth, and regulation of cell cycle, and mediation of apoptosis (73). The selective degradation or stabilization of intracellular proteins through the ubiquitin-dependent pathway is vital for adjusting the regulation of these cellular processes (73). Ubiquitination targets many key regulatory proteins for degradation of these proteins through the 26S proteasome. The NF-κB pathway proteins, p53, and the inhibitors of apoptosis (IAPs) proteins are well-known target of the proteasome (74). Due to these reasons, the proteasome acts as an attractive target for cancer therapeutics. Proteasome inhibitors are a novel class of compounds with promising anticancer effects. Proteasome inhibitors are more selective for cancer cells opposite to normal cells with the unknown reasons (75). Proteasome inhibitors also show additive effects in chemosensitization and radio sensitization of tumor cell lines (76). Proteasome inhibitor PS-341 sensitizes HCT-116 and HC4 cell lines to TRAIL through DR5 up-regulation and activation of extrinsic and intrinsic apoptotic pathway (77). MG132 also sensitizes HCT-116 cells to TRAIL by DR5 up-regulation (78). Bortezomib sensitizes acute myeloid leukemic cells to TRAIL by down-regulation of antiapoptotic proteins Bcl-xL and Mcl-1 and up-regulation of death receptors DR4, DR5, and proapoptotic protein p21, activation of executioner caspases, and a loss of the mitochondrial membrane potential (79). Bortezomib also sensitizes non-small lung carcinoma cells to TRAIL mediated apoptosis through DR5 up-regulation involving both the extrinsic and intrinsic apoptotic pathways (80). b-AP15, a novel inhibitor of proteasome deubiquitinating activity, sensitizes tumor cells to TRAIL mediated apoptosis through DR5 up-regulation and c-FLIP down-regulation (81). Proteasome inhibitor, NPI-0052, sensitizes tumor cells to TRAIL induced apoptosis by inhibiting the transcription repressor Yin Yang 1 (YY1), which regulates TRAIL resistance through the negative regulation of DR5. NPI-0052 up-regulates the DR5 promoter activity along with increase in both surface and total DR5 protein expression (82). The degradation of specific cell proteins is involved in determining cell proliferation or cell death. Inhibition of the ubiquitin–proteasome system by proteasome inhibitors blocks the process of programed cell death in thymocytes and neurons, but induces apoptosis in various human cancer cell lines (83). The ubiquitin–proteasome pathway has been also reported to control TRAIL apoptosis signaling by affecting levels of death domain adaptor molecules including Fas-associated death domain (FADD) and Fas-like inhibitor protein (FLIP) (73). Ubiquitination is considered to be a crucial regulator of DISC activity through recruitment of E3 ligase Cullin3 to the DISC. This recruitment leads to poly-ubiquitination of caspase-8 which results in DISC recruitment of the ubiquitin-binding protein p62, leading to the stabilization of the activated caspase-8, there by facilitating DISC activation (84). Inhibitors of apoptosis proteins (IAPs) are a family of proteins defined by the baculovirus repeat (BIR) domains and inhibit caspase activation; the majority of the caspase-inhibiting IAPs possess a carboxyl-terminal RING zinc-finger motif and exhibit E3 ligase activity (85). The overexpression of cIAP1 results in its autoubiquitination and degradation (86). cIAP2 can encourage monoubiquitination of caspase-3 and -7, and that XIAP catalyzes the ubiquitination and degradation of caspase-3 (87). This is supported by the study that IAPs catalyzed their own ubiquitination *in vitro*, and this activity requires the RING domain (87). The proteasome inhibitor PS-341 enhances TRAIL killing by increasing the level of DR5 and DR4 receptors, thus increasing caspase-8 activation (77). Proteasome inhibitor MG132 and MG115 sensitizes hepatocellular carcinoma cells to TRAIL by suppressing caspase inhibitors and the AKT signaling pathway (83). The proteasome inhibitors MG132 or Bortezomib sensitize human malignant pleural mesothelioma cells to TRAIL induced apoptosis through Mcl-1 and Akt protein cleavages (88). Bortezomib-mediated proteasome inhibition also sensitizes TRAIL resistant HPV-positive HNSCC cells to TRAIL-induced cell death through both the extrinsic and intrinsic pathways of apoptosis (89). Death-associated protein kinase (DAPK2) is a modulator of TRAIL signaling and inhibition of the expression of DAPK2 results in phosphorylation of NF-κB and transcriptional activity, which leads to induction of NF-κB target genes including DR4 and DR5 (90). Collectively, these findings indicate that the combination of proteasome inhibitors and TRAIL could be a promising strategy for TRAIL sensitization.

## Heat Shock Proteins Mediated TRAIL Resistance

Heat shock proteins (Hsp) are a highly conserved group of intracellular proteins classified by molecular weight into groups of Hsp110, Hsp90, Hsp70, Hsp60, small molecular Hsps (<27 kDa), and ubiquitin (91, 92). Hsps are highly abundant cytosolic proteins and function as molecular chaperones. Hsp function is best explained under cellular stress condition like heat, hypoxia under which levels of Hsps are significantly amplified (93). Under these stress conditions, Hsps encourage cell proliferation by inhibiting protein aggregation and enhancing the proper folding of damaged proteins (94, 95). Hsps also play a crucial role in normal cells, especially Hsp70, and to some amount Hsp90 are essentially implicated in protein folding functions (93–95). Hsp70 does so by binding to newly synthesized peptides thereby inhibiting premature protein misfolding, whereas Hsp90 binds to proteins with unstable tertiary structures and hamper protein degradation. Hsp60 and Hsp27 both function in protein folding by making a complex that make use of ATP to form intramolecular interactions required for client protein folding (96, 97). Hsp70 and Hsp90 are also implicated in the DNA-binding activity and stability of mutant p53, thereby resulting in cellular transformation (98). These findings indicate that the usual protein folding functions of Hsps, and in particular Hsp70 and Hsp90, are subverted by tumors to stabilize proteins important for the establishment and preservation of the transformed phenotype. Heat shock proteins such as Hsp70 and Hsp90 interact with Apaf-1, while Hsp27 sequesters cytochrome *c* from the cytoplasm, thereby preventing formation of apoptosome (99, 100). The heat shock proteins i.e., Hsp60 and Hsp10 promote procaspases three maturation (101), while Hsp90α has been reported to recruit FLIPs to DISC leading to TRAIL resistance. Inhibition of Hsp90 function affects multiple oncogenic substrates simultaneously and has been reported to have a TRAIL sensitizing effect (93). Combination of the Hsp90 inhibitor 17-AAG with “death receptor” targeting agents can synergistically improve their anti-tumor activities and decrease the TRAIL resistance in glioma cells (102). In TRAIL/TNF-resistant prostate cancer cells, pre- or co-treatment to17-AAG with TRAIL/TNF is known to induce high levels of apoptosis (103) through inhibition of the NF-κB or Akt cell survival pathways (104, 105). Synergistic effects between 17-AAG and anti-TRAIL monoclonal antibodies have also been observed (106). Collectively, these studies underscore the critical role of the Hsps in regulation of TRAIL resistance.

## Autophagy and TRAIL Resistance

Resistance to chemotherapeutic drugs is a universal clinical concern in cancer therapy. Intrinsic or acquired drug resistance can be due to a wide variety of mechanisms including tumor cell heterogeneity, drug efflux and metabolism, tumor microenvironment, or stress-induced genetic or epigenetic alterations as a cellular response to drug exposure (107). Among these mechanisms, the response or adaptation of cancer cell itself to anticancer drug-induced tumor microenvironment stresses is a fundamental cause for chemotherapy resistance. Autophagy is a lysosomal degradation process typically activated in response to adverse microenvironmental stresses (107). Autophagy itself fulfils a dual role, with both tumor-promoting and tumor-suppressing effects. Tumor cells activate autophagy in response to cellular stress and/or increased metabolic load related to enhanced cellular proliferation (108). As a response to anticancer treatments, whether autophagy activation leads to cell survival or cell death remains still unclear. Previous studies have suggested that the induction of autophagy could be a useful therapeutic approach to overcome drug resistance of cancers to some therapeutic agents, particularly those which typically induce an apoptotic response (107, 109). Cytoprotective autophagy circumvents TRAIL sensitivity, and inhibiting autophagy in cancer cells, sensitizes cancer cells to TRAIL (110). However, apoptosis-defective tumor cells can survive TRAIL-mediated stress by eliciting a protective autophagic process coupled with enhanced F-actin polymerization (88). Although the autophagy regulates key processes associated with TRAIL resistance, still more studies are needed to elucidate the molecular mechanisms of autophagy mediated TRAIL resistance and to provide basis for therapeutic approaches that can target autophagy mediated TRAIL resistance.

## Epigenetic Modulation and TRAIL Resistance

**Figure 3:**
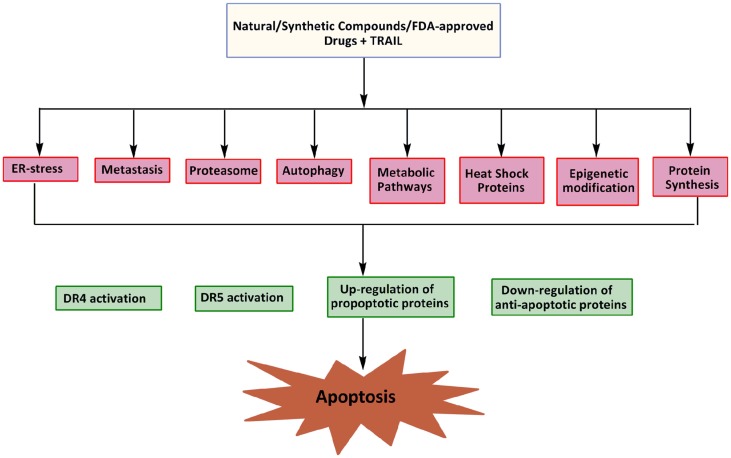
**Potential targets for sensitization of cancer cells to TRAIL induced apoptosis**.

Epigenetic changes may contribute to both cell survival and chemotherapy resistance in cancer cells. Abnormal DNA methylation at CpG islands and other associated epigenetic deregulations are observed during the acquisition of drug resistance (111). Recent studies suggest that epigenetic deregulation of gene expression by DNA methylation and aberrant histone deacetylation plays a crucial role in tumor development (112, 113). The role of epigenetic mechanisms in the silencing of the death receptor mediated pathway has been demonstrated in cases of medulloblastoma, as the inhibition of DNA methylation restored apoptosis suggesting the crucial role of DNA methylation in caspase-8 inactivation, a critical process in TRAIL resistance (114). Loss of caspase-8 gene expression critical to the process of TRAIL resistance by aberrant DNA methylation has also been supported by a number of other studies (114, 115). Overexpression of FAS/CD95 receptor and its cognate FAS ligand (FASL) are known to develop resistant in brain tumors toward etoposide treatment (116). The expression of DR4 and DR5 is also deregulated in human cancer cells by such mechanisms and it can be reversed by agents that target the DNA methylation or histone deacetylation (Figure 3). Modulation of chromatin by histone acetyltransferases (HATs) and histone deacetylases (HDACs) represents one important regulatory mechanism involved in gene transcription. Importantly, the HDAC inhibitor, MS-275 is effective in inhibiting the proliferation of cancer cells (Daoy and D283) *in vitro* through MS-275-induced increase in acetylation of histones H3 and H4 in the DR4 promoter and reactivation of DR4 expression in cancer cells. A significant potentiation of apoptosis observed in the presence of both MS-275 and recombinant TRAIL suggests that the up-regulated DR4 receptors are cell-surface associated and functional (111). At the receptor level, somatic mutations in the TRAIL receptors, down-regulation of DR4 and DR5, and over expression of the decoy receptors DcR1 and DcR2 can all confer selective resistance to TRAIL therapy (117). In summary, the reversal of aberrant gene repression with the use of a combination of epigenetic modulators and TRAIL could thus enhance the therapeutic benefit in a wide range of malignancies.

## Metabolic Pathways Involved in TRAIL Resistance

Metabolic processes and regulation in cancer cells differ significantly from the normal cells (158). Therefore, therapeutic targeting of metabolic pathways is a promising approach for enhancing TRAIL sensitivity in cancer cells. Cancer cells mostly rely on aerobic glycolysis, fatty acid synthesis, and glutaminolysis for their growth and proliferation (159). And this fact suggests that targeting cancer cell metabolism could provide a selective approach for targeting cancer cells without harming normal cells. Aerobic glycolysis or the Warburg effect links the high rate of anaerobic glycolysis to cancer (160). Mitochondrial respiration injury and hypoxia are often associated with resistance to chemotherapeutic drug-induced apoptosis. (161). One probable association between metabolic change and resistance to apoptosis is the association of HKs with the voltage-dependent channel protein (VDAC) under glycolytic metabolism. Tp53-induced glycolysis and apoptosis regulator (TIGAR), a target of p53, reduces the level of Fructose1, 6-biphosphatase, and ultimately inhibits glycolysis. TIGAR, a target of p53, inhibits glycolysis by reducing the level of FBP. Glucose is then diverted into the pentose phosphate pathway (PPP) to make NADH and nucleotides, instigating an increase in glutathione. As such, TIGAR decreases the sensitivity of cells to p53 and other apoptotic signals linked with ROS (162). Similarly, an over-expression of PFK redirects glucose from glycolysis to the PPP and increases the resistance to oxidative stress (163). This aberrant high rate of glycolysis generates microenvironmental acidosis which requires evolution to phenotypes resistant to acid-induced cellular toxicity. Following, cell populations with up-regulated glycolysis and acid resistance have a potent growth advantage, which stimulates unconstrained proliferation and cell invasion (164). Efforts have been made to adjust the metabolic reprograming of cancer cells by treating with glycolysis inhibiting compounds. 2-Deoxyglucose (2-DG) is best known as an inhibitor of glucose metabolism. Inside a cell, it is converted to phosphorylated 2-DG (2-DG-P) by hexokinase, the first and the rate-limiting enzyme in glycolysis (165). Glycolysis inhibitor, 2-DG, accumulates in cells and inhibits Hexokinase (HK). At high concentration, 2-DG leads to depletion of ATP level and results in apoptosis (166). 2-DG has been reported to sensitize tumor cells to death receptor induced apoptosis linking glucose metabolism to Mcl-1 down expression (151). The therapeutic potential of 2-DG has prompted sufficient interest in the United States, and there is an ongoing phase one clinical trial for this compound (ClinicalTrials.gov identifier: NCT00247403) (165). Dichloroacetate (DCA), another glycolysis inhibitor, prevents pyruvate dehyrogenase kinase (PDK) by increasing mitochondrial metabolism through forcing pyruvate in to mitochondria (167). DCA also reduces tumor growth *in vitro* and *in vivo* without affecting normal tissue (168). Altered expression of PKM2 is associated with drug resistance in different tumor. This shows that PKM2 is a potential target for adjuvant cancer therapy (169). Silencing of PKM2 intensify the efficacy of docetaxel because of enhanced inhibition of proliferation and apoptosis-inducing activity both *in vitro* and *in vivo* (170). It has been also reported that glucose deprivation intensifies TRAIL-induced apoptosis by decreasing the expression of cFLIP through the ceramide-AKT-FLIP pathway (171). Tumor cells tend to have a large pool of glutamate, and this pool is maintained by their ability to convert glutamine into glutamate through glutamine synthase (GLS), a mitochondrial enzyme highly active in tumors. Like glycolysis, this abnormal glutamine metabolism cancer cells makes these cells addicted to glutamine and this leads to increased synthesis of by-products essential for fast cell growth and proliferation (172, 173). Similarly, the salvage pathway of nucleotide synthesis is one of the attractive targets for cancer therapy. Dipyridamole is a known nucleoside transport inhibitor that sensitizes cancer cells to TRAIL induced apoptosis. Thymidylate synthase (TS) is an E2F-1 regulating enzyme, crucial for DNA synthesis and repair. Many cancer cells show elevated expression of this enzyme and have been associated with poor prognosis in various solid cancers including non-small cell lung cancer (174). The novel thymidylate synthase inhibitor trifluorothymidine (TFT) has been reported to enhance TRAIL-induced apoptosis in NSCLC cells by sensitizing the apoptotic machinery at different levels in the TRAIL pathway (154). The mitochondrion is the main power station of the cell that generates most of the cell’s supply of ATP by glycolysis and oxidative phosphorylation. In addition, mitochondria are also involved in a range of intracellular processes, such as cell growth and division, differentiation, apoptosis, and intracellular signaling. Mitochondria participate in *de novo* biosynthesis of pyrimidines, which is catalyzed by dihydroorotate dehydrogenase (DHODH), an FMN flavoprotein in the inner mitochondrial membrane, which transfers electrons from dihydroorotate to ubiquinone of the ETC for further oxidation (175). Doxorubicin is reported to sensitize cancer cells to TRAIL mediated apoptosis by targeting dihydroorotate dehydrogenase (DHODH) (155). Therefore, the novel inhibitors of metabolic pathways may be promising agents for TRAIL sensitization.

## Role of Metastasis in TRAIL Resistance

The process of cancer metastasis involves tumor cell invasion at the primary tumor, intravasation, arrest, and extravasation of the circulatory system, followed by angiogenesis and progressive outgrowth at a distant site (176, 177). Metastatic potential is measured by the number and size of large lesions on imaging and by indices of patient survival. Epithelial–mesenchymal transition (EMT) plays an essential role in promoting metastasis in epithelium-derived carcinoma and considered to be the key process driving tumor cell invasiveness and metastasis (178, 179). Recent studies have established the dynamic association of EMT and its reverse program, mesenchymal–epithelial transition (MET), in the metastatic process (179). EMT is characterized by the down-regulation of epithelial proteins, such as E-cadherin, γ-catenin/plakoglobin, α-catenin, and β-catenin (180) and with a stimulation of mesenchymal proteins, including α-smooth muscle actin, fibronectin, *N*-cadherin, or vimentin (181). This is mediated by transcription factors like Twist, E12/E47, and members of the Snail, and ZEB protein families (182, 183). ZEB1 suppresses E-cadherin expression by recruiting HDAC. EMT transition has been a novel target for TRAIL sensitization, and HDAC inhibitor MS-275 inhibits angiogenesis, reverses EMT, attenuates metastasis, and sensitizes TRAIL-resistant breast cancer MDA-MB-468 xenografts *in vivo* (178). A recent study has demonstrated that the increased migration and invasion is a crucial factor in regulation of TRAIL resistance in cancer cells (184). A recent study suggests that synergistic co-targeting of oncogenic and death receptor pathways can not only overcome melanoma resistance to different anti-tumor agents *in vitro*, but can also promote pro-apoptotic effects and inhibition of tumor angiogenesis *in vivo* (185). These data collectively support that metastatic potential of cancer cells can be a possible target for TRAIL sensitization.

## Therapeutic Targeting of TRAIL Resistance

Although TRAIL has high specificity and therapeutic efficacy against cancer cells, the mechanisms involved in TRAIL resistance are not well elucidated. Therefore, recent research efforts have focused on devising strategies to overcome TRAIL resistance in cancer cells in the clinical setting. A prolonged exposure at high concentrations of TRAIL might be required to overcome resistance (186, 187). However, the short plasma half life of TRAIL (7) due to rapid elimination through metabolism (6), achieving prolonged exposure at high concentrations is difficult. Recombinant TRAIL developed by Genentech (San Francisco, CA, USA) and Amgen (Thousand Oaks, CA, USA) is a receptor agonist that directly activates the functional death receptor TRAIL-R1 and TRAIL-R2, and used as a targeted therapy for both hematological malignancies and solid tumors. Pre-clinical studies have been performed using recombinant rhTRAIL and have provided evidence for the use of exogenous TRAIL for suppressing tumor growth both *in vitro* and *in vivo* (188, 189). A recent study indicated that the use of non-tagged version of rhTRAIL induces apoptosis in malignant cells but not in normal cells (190). It is also proven that non-tagged native rhTRAIL can reduce tumor growth without damaging human hepatocytes in the chimeric mouse model (191). Monoclonal antibodies targeting DR4 and DR5 have been proven to be clinically effective for cancer treatment because they can selectively bind to specific antigens and have longer half life as compared to rhTRAIL ligands (188). In 2008, a study reported the humanized DR5 agonistic MAb, CS-1008 generated from mouse DR5 MAb TRA-8 through a complementarity-determining region grafting (192) (Table 2). HGS-ETR1 (Mapatumumab; Human Genome Sciences, Rockville, MD, USA) is a fully human agonistic monoclonal antibody that binds TRAIL-R1, and it is in phase-II clinical trial as a single agent in patients with non-small cell lung cancer and colorectal cancer (193). Monoclonal antibodies target distinct receptor expression profile in malignant cells, whereas soluble TRAIL interacts with TRAIL-R1 and TRAIL-R2 as well as the decoy receptors. Therefore, soluble TRAIL may either have a wider therapeutic spectrum or a narrower and more unpredictable therapeutic window compared to that of the highly specific antibodies (194). The efficacy of selective drugs involved in TRAIL sensitization (Table 3) and survival profile of selective genes involved in TRAIL sensitization (Table 4) have been also identified using DRUGSURV and PPISURV respectively.

**Table 2:** **Summary of current recombinant human TRAIL variants, agonistic DR4, DR5-specific antibodies, their pre-clinical development and current clinical status**.

Molecule tested	Targeted receptors	Comments and clinical development
His-TRAIL (rhTRAIL variant)-polyhistidine-tagged rhTRAIL	DR4/DR5/decoy receptors	Induces apoptosis in transformed cells. Toxic to primary hepatocytes and keratinocytes (5, 195, 196)
LZ-TRAIL (rhTRAIL variant)-Leucin-zipper tagged rhTRAIL	DR4/DR5/decoy receptors	Induces apoptosis in transformed cell lines. Toxic to keratinocytes (6, 196)
Flag-TRAIL/M2 (rhTRAIL variant)	DR4/DR5/decoy receptors	On cross linking, induces apoptosis in transformed cells. Toxic to primary hepatocytes and keratinocytes (6, 11)
Apo2L/TRAIL (rhTRAIL variant)-non-tagged rhTRAIL	DR4/DR5/decoy receptors	Induces apoptosis in transformed cells, but not to primary, non-transformed hepatocytes, or keratinocytes. Ongoing phase I/II clinical trials as single agent and in combination therapy (7, 196–198) Amgen/Genentech
TRAIL-CD19 and TRAIL-EGFR (rhTRAIL fusion proteins)	DR4/DR5/Decoy receptors,TRAIL-CD19, and TRAIL-EGFR (rhTRAIL fusion proteins)	Selectively targets TRAIL to CD19 or EGFR expressing tumors, respectively. Induces apoptosis *in vitro*. Good *in vivo* activity seen with TRAIL-CD19 in pre-clinical studies (199, 200)
Apo2L.DR5–8 (rhTRAIL variant)	DR5/DcR2 (?)	Non-tagged, DR5-selective rhTRAIL variant. Induces apoptosis in DR5- responsive cancer cell lines. Toxicity observed following cross-linking (201)
DR5-TRAIL (E195R/D269H) (rhTRAIL variant)	DR5/DcR2 (reduced)	Non-tagged, DR5-selective rhTRAIL. Induces apoptosis in DR5-responsive cancer cell lines. No toxicity in non-transformed fibroblast and endothelial cells. Anti-tumor activity in ovarian cancer xenograft models (202)
M413 (agonistic Ab)	DR5	Induces apoptosis in TRAIL-sensitive cancer cell lines selectively through DR5 receptor (203)
TRA-8 (CS-1008) (agonistic Ab)	DR5	Induces apoptosis in DR5-responsive cancer cell lines and primary hepatocellular carcinoma but not toxic to normal hepatocytes (phase I clinical trials), (204) (Sankyo)
AMG 655 (agonistic Ab)	DR5	Induces apoptosis in a number of human cancer cell lines. Phase I trial showing dose linear kinetics with half-life of 10 days and some anti-tumor activity (Amgen)
LBY135 (agonistic Ab)	DR5	Good anti-tumor activity *in vitro* and *in vivo* pre-clinical studies. Currently in phase I trials (Novartis)
Lexatumumab (HGSETR2,agonistic Ab) HGS-TR2J (agonistic Ab)	DR5	Phase I/Ib trials showing that lexatumumab can be administered safely and in combination with chemotherapeutic agents. (Human Genome Science) (205). HGS-TR2J was voluntarily suspended from clinical development
Apomab (agonistic Ab)	DR5	Phase I trial showing dose proportional pharmacokinetics. Half-life 15–20 days. Currently initiations of phase II trial (Genentech) (206)
TRAIL-R1-5 (rhTRAIL variant)	DR4/decoy receptors (?)	Non-tagged, DR4-selective rhTRAIL. Induces apoptosis in DR4 responsive cancer cell lines. HDACi sensitized primary CLL cells to DR4 mediated apoptosis (207)
M271 (agonistic Ab)	DR4	Induces apoptosis in TRAIL-sensitive cancer cell lines selectively through DR4 receptor (203)
4HG, 4G7 (agonistic Ab)	DR4	Induced apoptosis *in vitro* with cross-linking antibody. Anti-tumor activity in colon cancer xenograft model (208)
2E12 (agonistic Ab)	DR4	Induced apoptosis *in vitro* with cross-linking antibody (204)
Mapatumumab (HGS-ETR1) (agonistic Ab)	DR4	Phase I – solid malignancies refractory to standard therapy, safely administered up to 10 mg/kg
		Phase Ib – combination therapy with paclitaxel and carboplatin (209)
		Phase II – single treatment in NSCLC

**Table 3:** **The efficacy of selective drugs involved in TRAIL sensitization (212)**.

Drugs	Direct targets					Indirect targets				
	Cancer type	*P*-value, FDR adjusted	Odds ratio	*k* (*l*)	*m* (*N*)	Cancer type	*P*-value, FDR adjusted	Odds ratio	*k* (*l*)	*m* (*N*)
Bortezomib	Breast cancer	0.23 (0.0085)	4.55	4 (11)	1295 (16154)	Breast cancer	0.045 (0.00102)	2.23	18 (93)	2127 (24375)
	Glioblastoma	0.23 (0.011)	6.16	3 (11)	575 (12940)	Lung cancer	0.047 (0.0021)	2.03	19 (92)	2002 (19592)
	Diffuse large B cell lymphoma	0.65 (0.046)	2.05	6 (11)	5432 (20387)	Diffuse large B cell lymphoma	0.069 (0.0059)	1.47	36 (92)	5432 (20387)
Valproic acid	Glioblastoma	0.086 (0.0020009)	6.97	4 (13)	575 (12940)	Diffuse large B cell lymphoma	0.0037 (8.57e −05)	1.64	48 (110)	5432 (20387)
	Breast cancer	0.41 (0.019)	2.96	5 (13)	1685 (12940)	Breast cancer	0.41 (0.024)	1.90	12 (96)	858 (12940)
	Breast cancer	0.44 (0.031)	4.23	3 (14)	1278 (25177)	Breast cancer	0.41 (0.033)	1.68	15 (112)	1295 (16154)
Anisomycin	Breast cancer	0.0104 (0.00023)	5.34	7 (26)	1034 (20387)	Diffuse large B cell lymphoma	0.00012 (2.86e −06)	1.42	137 (365)	5432 (20387)
	Breast cancer	0.025 (0.0011)	4.86	6 (26)	973 (20387)	Chronic lymphocytic leukemia	0.00039 (1.80e −05)	1.70	66 (365)	2200 (20386)
	Astrocytic glioma	0.24 (0.018)	5.26	3 (20)	535 (18681)	Breast cancer	0.0040007 (0.00036)	1.65	51 (345)	1180 (12940)
						Ovarian cancer	0.0040007 (0.00038)	1.92	33 (345)	660 (12940)
						Breast cancer	0.0040007 (0.00045)	1.62	52 (371)	2127 (24375)
						Breast cancer	0.018 (0.0025)	1.72	32 (371)	1278 (25177)
						Astrocytic gliomas	0.034 (0.00609)	1.97	18 (325)	535 (18681)
						High-grade glioma	0.034 (0.0063)	1.52	40 (345)	1000 (12940)
						Multiple myeloma	0.0403 (0.0082)	1.51	38 (365)	1416 (20387)
						Breast cancer	0.043 (0.0099)	1.47	41 (352)	1295 (16154)
Doxorubicin	Breast cancer	0.16 (0.0036)	3.55	6 (13)	1685 (12940)	Diffuse large B cell lymphoma	1.56e −05 (3.56e −07)	1.52	116 (289)	5432 (20387)
	Diffuse large B cell lymphoma	0.19 (0.011)	2.11	9 (16)	5432 (20387)	Chronic lymphocytic leukemia	4.89e −05 (2.22e −06)	1.88	58 (289)	2200 (20386)
	Chronic lymphocytic leukemia	0.19 (0.016)	3.15	5 (16)	1844 (18540)	Breast cancer	0.083 (0.0057)	1.44	49 (263)	1685 (12940)
Trifluorothymidine	Breast cancer	0.26 (0.0083)	6.48	3 (7)	858 (12940)	Diffuse large B cell lymphoma	6.04e −16 (1.37e −17)	1.70	210 (472)	5432 (20387)
	High-grade glioma	0.26 (0.012)	5.56	3 (7)	1000 (12940)	Chronic lymphocytic leukemia	1.00e −06 (5.02e −08)	1.80	90 (472)	2200 (20386)
	Prostate cancer	0.43 (0.0307)	6.93	2 (6)	295 (6097)	Breast cancer	1.00e −06 (6.84e −08)	1.90	77 (473)	2127 (24375)
						Breast cancer	0.0033 (0.000304)	1.79	42 (472)	1034 (20387)
						Lung cancer	0.0067 (0.00076)	1.47	71 (477)	2002 (19592)
						Breast cancer	0.0082 (0.0011)	1.69	40 (473)	1278 (25177)
						Breast cancer	0.0082 (0.0014)	1.40	80 (445)	1685 (12940)
						Breast cancer	0.0082 (0.0014)	1.53	54 (446)	1295 (16154)
						Lung cancer	0.0087 (0.0017)	2.24	18 (472)	357 (20387)
						Glioblastoma	0.012 (0.0027)	1.71	33 (445)	575 (12940)
Decitabin	Liposarcoma	0.99 (0.054)	4.73	2 (3)	1827 (12940)	Liposarcoma	0.0049 (0.00011)	3.40	11 (23)	1827 (12940)
	Breast cancer	0.99 (0.14)	6.58	1 (3)	1034 (20387)	Breast cancer	0.12 (0.0056)	3.09	7 (26)	2127 (24375)
	Multiple myeloma	0.99 (0.19)	4.80	1 (3)	1416 (20387)	Breast cancer	0.15 (0.012)	3.00	6 (25)	1295 (16154)
Dipyridamole	Cervical cancer	0.75 (0.021)	3.67	4 (23)	688 (14453)					
	Ovarian cancer	0.75 (0.059)	5.06	2 (23)	224 (12940)	
	Diffuse large B cell lymphoma	0.75 (0.079)	1.56	10 (24)	5432 (20387)	

**k* the number of drug targets (genes) significantly associated (*P*-value <0.01) with survival in the dataset*.

**l* the overall number of known drug targets*.

**m* the overall number of genes significantly associated with survival in the dataset*.

**N* the overall number of genes measured in the dataset*.

**Table 4:** **Survival profile of selective genes involved in TRAIL sensitization (212)**.

Gene	Cancer type	GENE (probe ID)	*P*-value	Effect sign
DDIT3	Breast cancer	209383_AT	2.4e-05	Negative
	Lung cancer	209383_AT	0.00318	Negative
	Breast cancer	11002	0.00765	Negative
	Ovarian cancer	209383_AT	0.0326	Positive
	Cervical cancer	CG15021531	0.068	Negative
	Astrocytic gliomas	956	0.0725	Negative
	Colon cancer	209383_AT	0.0774	Negative
	Lung cancer	209383_AT	0.0799	Negative
	Bladder cancer	ILMN_1676984	0.149	Negative
	Breast cancer	209383_AT	0.232	Positive
	Multiple myeloma	209383_AT	0.438	Positive
	Breast cancer	A_23_P21134	0.533	Positive
	Breast cancer	22873	0.667	Positive
	Chronic lymphocytic leukemia	209383_AT	0.701	Positive
	Breast cancer	209383_AT	0.845	Positive
	Chronic lymphocytic leukemia	209383_AT	0.869	Positive
p53	Breast cancer	201746_AT	0.00691	Positive
	Multiple myeloma	201746_AT	0.0106	Positive
	Breast cancer	211300_S_AT	0.0145	Positive
	Diffuse large B cell lymphoma	211300_S_AT	0.0289	Negative
	Breast cancer	211300_S_AT	0.0378	Positive
	Cervical cancer	CG11519508	0.0583	Positive
	High-grade glioma	201746_AT	0.115	Negative
	Lung cancer	18627	0.14	Negative
	Breast cancer	1330	0.14	Negative
	Lung cancer	A_23_P26810	0.147	Negative
	Breast cancer	ILMN_1779356	0.158	Negative
	Astrocytic gliomas	13689	0.165	Positive
c-myc	Diffuse large B cell lymphoma	202431_S_AT	9e-04	Negative
	Breast cancer	19825	0.0019	Negative
	Meningioma	302	0.0091	Negative
	Breast cancer	202431_S_AT	0.0181	Negative
	Breast cancer	A_23_P215956	0.0191	Positive
	Lung cancer	A_23_P215956	0.026	Negative
	High-grade glioma	202431_S_AT	0.0416	Positive
	Colon cancer	202431_S_AT	0.0576	Positive
	Breast cancer	202431_S_AT	0.0622	Positive
	Breast cancer	ILMN_1680618	0.0656	Negative
	Breast cancer	202431_S_AT	0.0666	Negative
	Lung cancer	8	0.0813	Negative
Cflar	Liposarcoma	209508_X_AT	0.000199	Positive
	Diffuse large B cell lymphoma	211316_X_AT	0.000565	Negative
	Chronic lymphocytic leukemia	237367_X_AT	0.00119	Negative
	Lung cancer	239629_AT	0.00121	Positive
	Multiple myeloma	211316_X_AT	0.00133	Positive
	Breast cancer	209939_X_AT	0.00171	Negative
	Breast cancer	208485_X_AT	0.00323	Positive
	Cervical cancer	CG18119407	0.00609	Negative
	Breast cancer	210563_X_AT	0.013	Positive
	Breast cancer	210563_X_AT	0.0138	Positive
TNFRSF10B	Breast cancer	209295_AT	0.000221	Positive
	Diffuse large B cell lymphoma	210405_X_AT	0.00271	Negative
	Lung cancer	210405_X_AT	0.0221	Negative
	Breast cancer	210405_X_AT	0.0241	Positive
	Multiple myeloma	209295_AT	0.0271	Positive
	Ovarian cancer	209295_AT	0.0551	Negative
	Breast cancer	210405_X_AT	0.062	Negative
	Breast cancer	16038	0.0684	Positive
	Chronic lymphocytic leukemia	210405_X_AT	0.0732	Negative
	Liposarcoma	209295_AT	0.0776	Positive
	High-grade glioma	209295_AT	0.0832	Negative
	Breast cancer	3130377	0.0869	Positive

Administration of soluble TRAIL in animal models of cancer has shown significant antitumor effect without any systemic toxicity. In *in vivo* studies, a trimerized (6) or a non-tagged (7, 191) form of TRAIL has shown a good toxicity profile, and organ toxicity might be expected at high doses of soluble TRAIL. In TRAIL related cancer therapeutics, recombinant soluble form of TRAIL, rhTRAIL (Dulanermin), the TRAIL R1-targeting agonistic monoclonal antibody mapatumumab; and the TRAIL R2-targeting agonistic monoclonal antibodies conatumumab, tigatuzumab, HGS-ETR-2 (lexatumumab), and DAB4 (PRO95780) against different types of cancer including non-Hodgekin lymphoma and colorectal cancer have been advanced to clinical development as chemotherapeutic agents (210). Mostly non-small cell lung carcinoma and other solid cancers are treated with a combination of rhTRAIL (Dulanermin) and paclitaxel, carboplatin and bevacizumab; mapatumumab and paclitaxel or carboplatin, as well as mapatumumab combined with gemcitabine or cisplatin (211). These combinations are evolving as very effective treatment against TRAIL resistant cancer cells.

## Conclusion and Future Prospects

In the last decade, search for novel cancer therapeutics has focused on the goal of developing specific, targeted, and less toxic agents for treatment of cancers. In this context, TRAIL as a promising chemotherapeutic agent has attracted much attention, and is currently being evaluated in the phase II clinical trials. However, the dogged pursuit of validating TRAIL as a specific anti-cancer agent has further highlighted its limitations in the clinical setting. The precise mechanisms involved in the escape from TRAIL-induced cytotoxicity and development of TRAIL resistance in some cancer cells is still not well understood. Whether combination of TRAIL receptor agonists with natural or synthetic TRAIL sensitizers will restore cancer cell sensitivity toward TRAIL is still an open question (213). It is still not well-understood whether the cellular processes alone or in combination can induce TRAIL resistance. It is also not known whether different types of tumor undergo TRAIL resistance through similar or specific mechanisms? The question whether and how TRAIL resistance could be measured to monitor therapy response in patients needs further attention. Pre-clinical studies till date suggest that combination therapy with TRAIL and chemotherapeutic drugs, natural compounds, or radiation is undoubtedly a logical way forwards in devising rationalized therapeutic regimens for TRAIL resistant cancers. However, effective therapeutic targeting of TRAIL resistance will essentially need to focus on (1) development of strategies for increasing the half life of TRAIL, (2) identification of suitable biomarkers through pre-selection of patients responsive to rhTRAIL/agonist antibody therapy, (3) development of novel synergistic combinations with TRAIL and inhibitors of cell stress response proteins, and (4) screening and identification of novel TRAIL sensitizers from FDA approved drug libraries. In the future, combination therapies with TRAIL would necessitate targeting the signaling pathways associated with the self-seeding properties of each cancer along with their varying pre-metastatic niches. However, given the complexity of the TRAIL system, further studies in primary tumor cells of diverse origin along with validation studies through syngenic and xenograft mice models and clinical trials would be required to develop personalized medicine on the basis of the TRAIL/TRAIL receptor pathway.

## Author Contributions

RT: collected and reviewed the literature and wrote the manuscript. DM: corrected and revised the manuscript. Both authors read and approved the final manuscript.

## Conflict of Interest Statement

The authors declare that the research was conducted in the absence of any commercial or financial relationships that could be construed as a potential conflict of interest.

## Author Note

Rachana Trivedi has received a Master of Technology (M.Tech) degree in Biotechnology from the West Bengal University of Technology, India, in 2008. In 2009, she received a CSIR-Junior Research Fellowship, funded by the Council for Scientific and Innovative Research, Government of India, and is currently pursuing a Ph.D program in Cancer Biology at the Endocrinology Division of the CSIR-Central Drug Research Institute, Lucknow, under the supervision of Dr. Durga Prasad Mishra.

Durga Prasad Mishra, Principal Scientist, is the Head of the Cell Death Research Laboratory (CDRL) at the Endocrinology Division of the CSIR-Central Drug Research Institute, Lucknow, India. He has received his master’s degree in Physiology and Ph.D in Molecular Endocrinology. The prime focus of his laboratory is to study the molecular mechanisms of cell death in cancer, neuronal, and male germ cells. CDRL is actively screening for TRAIL sensitizers using commercially available FDA approved drug libraries and natural compound derived libraries synthesized at the CSIR-Central Drug Research Institute.

## Abbreviations

c-FLIP, cellular FLICE-like inhibitory protein; DcR, decoy receptor; DD, death domain; DED, death effector domain; DISC, death inducing signaling complex; DR, death receptor; EMT, epithelial mesenchymal transition; HDAC, histone deacetylase; HSPs, heat shock proteins; JNK, c-Jun-*N*-terminal kinase; NF-κB, nuclear factor kappa B; OPG, osteoprotegerin; RANKL, receptor activator of nuclear factor kappa-B ligand; TNF, tumor necrosis factor; TRAIL, tumor necrosis factor-related apoptosis-inducing ligand; XIAP, X-linked inhibitor of apoptosis protein; UPR, unfolded protein response; UV, ultraviolet.
